# Does pregnancy complication history improve cardiovascular disease risk prediction? Findings from the HUNT study in Norway

**DOI:** 10.1093/eurheartj/ehy863

**Published:** 2018-12-27

**Authors:** Amanda R Markovitz, Jennifer J Stuart, Julie Horn, Paige L Williams, Eric B Rimm, Stacey A Missmer, Lauren J Tanz, Eirin B Haug, Abigail Fraser, Simon Timpka, Bjørnar Klykken, Håvard Dalen, Pål R Romundstad, Janet W Rich-Edwards, Bjørn Olav Åsvold

**Affiliations:** 1 Department of Epidemiology, Harvard T.H. Chan School of Public Health, 677 Huntington Ave, Boston, MA, USA; 2 Division of Women's Health, Department of Medicine, Brigham and Women's Hospital and Harvard Medical School, 1620 Tremont Street, Boston, MA, USA; 3 Mathematica Policy Research, 955 Massachusetts Avenue, Cambridge, MA, USA; 4 Department of Public Health and Nursing, NTNU, Norwegian University of Science and Technology, Postboks, N-7491 Trondheim, Norway; 5 Department of Obstetrics and Gynecology, Levanger Hospital, Nord-Trøndelag Hospital Trust, Kirkegata 2, Levanger, Norway; 6 Department of Biostatistics, Harvard T.H. Chan School of Public Health, 677 Huntington Ave, Boston, MA, USA; 7 Department of Nutrition, Harvard T.H. Chan School of Public Health, 677 Huntington Ave, Boston, MA, USA; 8 Channing Division of Network Medicine, Department of Medicine, Brigham and Women's Hospital and Harvard Medical School, 181 Longwood Ave, Boston, MA, USA; 9 Division of Adolescent and Young Adult Medicine, Department of Pediatrics, Boston Children's Hospital and Harvard Medical School, 333 Longwood Ave, Boston, MA, USA; 10 Department of Obstetrics, Gynecology, and Reproductive Biology, College of Human Medicine, Michigan State University, 400 Monroe Ave. NW, Grand Rapids, MI, USA; 11 Population Health Sciences, Bristol Medical School and MRC Integrative Epidemiology Unit at the University of Bristol, Oakfield House, Oakfield Grove, UK; 12 Harvard Medical School, 25 Shattuck St., Boston, MA, USA; 13 Lund University Diabetes Center, Department of Clinical Sciences, Malmö, Lund University, Jan Waldenströms gata 35, Malmö, Sweden; 14 Department of Medicine, Levanger Hospital, Nord-Trøndelag Hospital Trust, Kirkegata 2, Levanger, Norway; 15 Department of Circulation and Medical Imaging, NTNU, Norwegian University of Science and Technology, Postboks 8905, Trondheim, Norway; 16 Department of Cardiology, St. Olavs Hospital, Trondheim University Hospital, Prinsesse Kristinas gate 3, Trondheim, Norway; 17 K.G. Jebsen Center for Genetic Epidemiology, Department of Public Health and Nursing, NTNU, Norwegian University of Science and Technology, Postboks 8905, Trondheim, Norway; 18 Department of Endocrinology, St. Olavs Hospital, Trondheim University Hospital, Prinsesse Kristinas gate 3, Trondheim, Norway

**Keywords:** Prediction, Stroke, Coronary heart disease, Pregnancy, Women’s health

## Abstract

**Aim:**

To evaluate whether history of pregnancy complications [pre-eclampsia, gestational hypertension, preterm delivery, or small for gestational age (SGA)] improves risk prediction for cardiovascular disease (CVD).

**Methods and results:**

This population-based, prospective cohort study linked data from the HUNT Study, Medical Birth Registry of Norway, validated hospital records, and Norwegian Cause of Death Registry. Using an established CVD risk prediction model (NORRISK 2), we predicted 10-year risk of CVD (non-fatal myocardial infarction, fatal coronary heart disease, and non-fatal or fatal stroke) based on established risk factors (age, systolic blood pressure, total and HDL-cholesterol, smoking, anti-hypertensives, and family history of myocardial infarction). We evaluated whether adding pregnancy complication history improved model fit, calibration, discrimination, and reclassification. Among 18 231 women who were parous, ≥40 years of age, and CVD-free at start of follow-up, 39% had any pregnancy complication history and 5% experienced a CVD event during a median follow-up of 8.2 years. While pre-eclampsia and SGA were associated with CVD in unadjusted models (HR 1.96, 95% CI 1.44–2.65 for pre-eclampsia and HR 1.46, 95% CI 1.18–1.81 for SGA), only pre-eclampsia remained associated with CVD after adjusting for established risk factors (HR 1.60, 95% CI 1.16–2.17). Adding pregnancy complication history to the established prediction model led to small improvements in discrimination (C-index difference 0.004, 95% CI 0.002–0.006) and reclassification (net reclassification improvement 0.02, 95% CI 0.002–0.05).

**Conclusion:**

Pre-eclampsia independently predicted CVD after controlling for established risk factors; however, adding pre-eclampsia, gestational hypertension, preterm delivery, and SGA made only small improvements to CVD prediction among this representative sample of parous Norwegian women.

**Figure:**
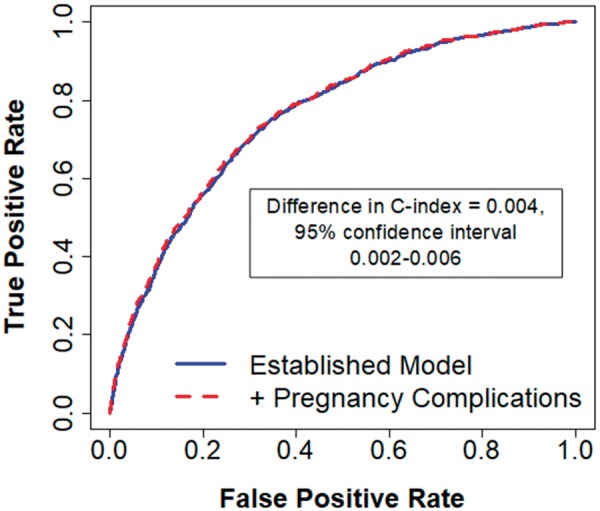

**See page 1121 for the editorial comment on this article (doi: 10.1093/eurheartj/ehz021)**

## Introduction

Cardiovascular disease (CVD) is the leading cause of death in women^1^ and is underdiagnosed in women compared to men.^2^^,^^3^ Cardiovascular disease risk prediction tools are commonly used to identify individuals at higher risk, allowing physicians to target interventions to patients who would benefit the most. However, clinical guidelines have noted that current tools identify few high risk women prior to age 70 and have called for research to improve sensitivity for younger women.^4^^,^^5^

Pregnancy may provide an ideal ‘window’ to predict future cardiovascular health in young women.^6^^,^^7^ Emerging evidence suggests that pregnancy complications, including pre-eclampsia,^8^^,^^9^ gestational hypertension,^10^ preterm delivery,^11^^,^^12^ and delivery of an infant small for gestational age (SGA),^13–15^ are associated with future CVD. Associations between pregnancy complications and CVD are likely due to shared etiologic pathways^7^ (e.g. metabolic syndrome, vascular dysfunction, and inflammation). Current guidelines recommend screening women for pregnancy complications and monitoring cardiovascular risk factors postpartum.^2^^,^^16^^,^^17^ However, it is unclear whether pregnancy complications also have utility in predicting CVD above-and-beyond established risk factors already included in prediction models. There is some evidence that pregnancy complications do not lead to large improvements in CVD risk prediction.^18^^,^^19^ However, only one previous study examined the benefit of adding pregnancy complications to existing CVD prediction models in a clinical setting,^18^ and no published studies have examined the benefit of adding history of preterm delivery or SGA to existing models.

Using linked data from the Nord-Trøndelag Health Study (the HUNT Study), the Medical Birth Registry of Norway (MBRN), validated hospital records, and the Norwegian Cause of Death Registry, we evaluated whether adding history of pregnancy complications to the NORRISK 2 risk prediction model,^20^ the model currently recommended for use in clinical practice in Norway,^21^ improved prediction performance in parous women.

## Methods

### Study population

The HUNT Study is an ongoing population-based cohort study of residents in Norway’s Nord-Trøndelag county. Approximately every decade, all county residents 20 years of age and older are invited to participate in an extensive health assessment, including a clinical examination and questionnaires.^22^ Three surveys were completed by the time of this analysis, HUNT1 (1984–86),^23^ HUNT2 (1995–97),^24^ and HUNT3 (2006–08).^22^ Using the national identification number assigned to Norwegian citizens, we linked HUNT data to the MBRN^25^ to capture information about all deliveries that occurred after the birth registry began in 1967.

**Figure 1:**
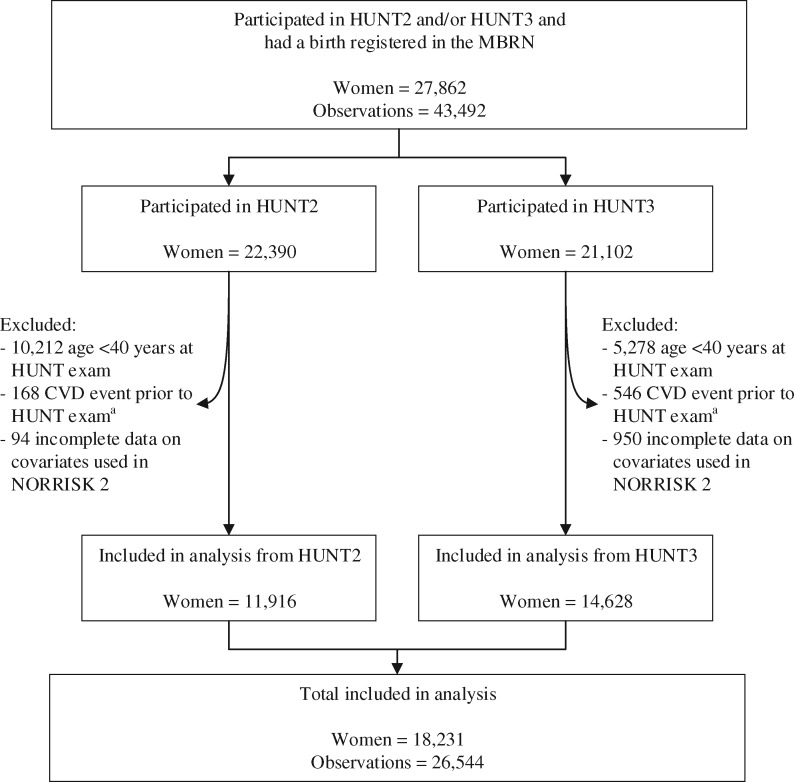
Flow chart of study population. ^a^Includes self-reported history of myocardial infarction or stroke at HUNT exam and hospitalizations for myocardial infarction or stroke recorded from 1987 through the date of HUNT exam. CVD, cardiovascular disease; HUNT, the Nord-Trøndelag Health Study; MBRN, Medical Birth Registry of Norway.

We restricted this analysis to participants in the HUNT2 and HUNT3 surveys, during which serum samples were collected for all participants, to enable inclusion of lipids in CVD risk prediction models. We identified a total of 27 862 parous women who participated in HUNT2 and/or HUNT3 and had a birth registered in the MBRN (*Figure 1*). As the NORRISK 2 CVD risk prediction model^20^ included only adults ages 40 years and older, we excluded women younger than 40 years at the time of the HUNT exam (*n* = 9056). We also excluded women with a history of CVD prior to the HUNT exam (*n* = 292). History of CVD was identified through either (i) self-report of myocardial infarction (MI) or stroke via questionnaire during the HUNT exam or any previous HUNT exam (including HUNT1) or (ii) validated record of hospitalization for MI or stroke from the start of record collection in 1987 through the date of the HUNT exam. After additionally excluding women with incomplete data on covariates used in the NORRISK 2 prediction model^20^ (*n* = 283), the final study population included 18 231 women. All HUNT study participants signed an informed consent form permitting use of their data and samples for research. The Central Norway Regional Committee for Medical and Health Research Ethics approved this project, and the Harvard T. H. Chan School of Public Health exempted this project from IRB review.

### Established cardiovascular risk factors

Established risk factors [including systolic blood pressure, total cholesterol, high density lipoprotein cholesterol (HDL-C), smoking, anti-hypertensive use, and family history of premature MI] were chosen to match the NORRISK 2^20^ model to the extent possible. Trained HUNT study staff measured systolic blood pressure and collected non-fasting serum samples to quantify total cholesterol and HDL-C. We defined low HDL-C as <1.3 mmol/L. Supplementary material online*, Table S1* includes additional measurement details. From the HUNT questionnaires, we identified current daily smoking and current anti-hypertensive use at the time of the HUNT exam as well as family history of premature MI, defined as having a first-degree family member who suffered a MI before the age of 60 years.

### Pregnancy complications

We identified pregnancy complication history from the MBRN, including all pregnancies from 1967 to the time of the HUNT exam. Diagnoses of pre-eclampsia or gestational hypertension used internationally recommended criteria,^26^^,^^27^ with gestational hypertension generally defined as *de novo* hypertension (≥140 mmHg systolic and/or ≥90 mmHg diastolic) after 20 weeks of gestation, and pre-eclampsia also requiring proteinuria (300 mg/24 h or ≥1+ on the dipstick test). We identified gestation length based on ultrasound dating where available (2% of deliveries) or last menstrual period and defined preterm delivery as <37 weeks gestation. Multiple gestational pregnancies delivered <37 weeks were excluded from the preterm delivery definition. Small for gestational age was defined as the lowest 10% of birthweights by gestational age and sex observed in the MBRN,^28^ with percentiles calculated separately for multiple vs. single gestational pregnancies. Validation studies within HUNT^26^^,^^29^ found positive predictive values (PPV) of 88% for pre-eclampsia and 93% for preterm delivery. Gestational hypertension’s PPV was 68%, due in part to mislabeling of pre-eclampsia cases as gestational hypertension. Among women classified as gestational hypertensives, 88% had a hypertensive disorder of pregnancy (HDP). Although SGA was not included in validation studies, the PPV for low birth weight (<2500 g) in the HUNT study was 100%. Pregnancy complications were analysed using separate variables indicating whether the women had a history of the complication of interest across any pregnancy.

### Cardiovascular endpoints

Consistent with NORRISK 2, we predicted hard CVD endpoints (non-fatal MI, fatal CHD, or non-fatal or fatal stroke). CVD events were captured from the two primary hospitals in Nord-Trøndelag county: Levanger Hospital and Namsos Hospital (Nord-Trøndelag Hospital Trust) from 1, September 1987 (the beginning of electronic recording) through 24 April 2015. All study participants with at least one cardiovascular diagnosis had their medical records reviewed by one of the two experienced cardiologists (B.K. and H.D.) to determine the first validated occurrence of MI or stroke. We diagnosed MI using Joint ESC/ACCF/AHA/WHF Task Force criteria^30^ and stroke based on typical symptoms and signs combined with radiological evidence from CT or MRI scans. Supplementary material online, *Text S1* includes additional validation process details. In addition, we identified fatal CVD events using ICD diagnostic codes from the national Cause of Death Registry, which has had mandatory reporting since 1951 (Supplementary material online, *Table S2*).

### Statistical analysis

We sought to compare an established CVD risk factor model to that same model additionally including history of pregnancy complications. For each of these models, we used the same statistical methods as NORRISK 2.^20^ We estimated the 10-year risk of CVD using Fine and Gray competing risk models,^31^ accounting for deaths from other causes as competing events. Women contributed person-time to the analysis from the index HUNT exam through first CVD event, death from other causes, or censoring at either the end of data collection on 24 April 2015 or emigration from Nord-Trøndelag county. Women who participated in both the HUNT2 and HUNT3 exams (*n* = 8313) were allowed to contribute two independent (non-overlapping) observations to the analysis as long as they met eligibility criteria at the start of follow-up. These women could contribute an observation with follow-up (i.e. at the time of the exam) from HUNT2 to HUNT3 using risk factor information assessed at the time of HUNT2 and an observation with follow-up from HUNT3 using risk factor information assessed during HUNT3. Although women could have contributed more than 10 years of follow-up to the analysis based on the timing of exams, we predicted CVD risk at 10 years of follow-up. To account for the correlation in measurements among women who participated in HUNT2 and HUNT3, we used variance estimates which account for repeated measures^32^ (Supplementary material online, *Text S2*). Some pregnancies were missing from the registry due to women giving birth before the start of the birth registry in 1967 (*n* = 4387 women), and some pregnancies had missing values for gestation length and/or birthweight or pregnancies with Z-scores for birthweight by gestation length that were >4 or <−4, suggesting error during data entry (*n* = 2042 women). We used multiple imputation to impute pregnancy complications for these pregnancies missing from the registry or with incomplete information in the registry. Supplementary material online, *Text S2* provides details about multiple imputation methods used.

We calculated measures of model fit, calibration, discrimination, and reclassification because no single measure captures all of the information needed to assess improvement in model performance after adding pregnancy complications.^33^ Prior to building models, we compared the established risk factors between those with and without a history of pregnancy complications and tested whether there were significant differences using either χ^2^ tests for categorical measures or Student’s *t*-tests for continuous measures. We then estimated an unadjusted model including only pregnancy complications to confirm their ability to predict CVD endpoints in our population. Next, we identified whether the addition of pregnancy complications to the established risk factor model improved model fit using a Wald test. We assessed model calibration (the equivalence between observed CVD risk and model-predicted CVD risk) for models using the Greenwood–Nam–D’Agostino test for censored survival data.^34^ We measured model discrimination (the ability to distinguish between CVD cases and non-cases) by obtaining the C-index for each model and comparing the difference between them.^35^ The C-index is an extension of the concept of the area under the receiver operating characteristic (ROC) curve but modified to be appropriate for the survival setting.

A key indicator of improved model performance is if, after adding pregnancy complications, women who went on to have a CVD event were reclassified into higher risk categories while women who did not have an event were reclassified into lower risk categories.^36^ This information is summarized in the net reclassification improvement (NRI) which we calculated overall and separately among women who did and did not go on to have a CVD event, using an extension of the formula for survival data.^37^ To calculate these measures, we stratified women into clinically relevant categories based on their 10-year risk of CVD: low (<5%), intermediate (5 to <10%), and high (≥10%). We also calculated the integrated discrimination improvement (IDI),^36^ which is a related measure of model improvement that does not rely on cut-points to categorize risk. Confidence intervals for discrimination and reclassification measures were calculated using 1000 bootstraps. All analyses were performed using SAS version 9.4 (SAS Institute Inc., Cary, NC, USA) and used publically available macros to assess prediction performance.^38^ See Supplementary material online, *Text S**2* for additional details about prediction performance measures.

### Additional analyses

In *post hoc* analyses suggested by reviewers, we included additional terms for recurrent pregnancy complications (i.e. for each pregnancy complication, having experienced the complication during two or more pregnancies) and interaction terms between the pregnancy complications to test alternative parameterizations for modelling pregnancy complication history. As a sensitivity analysis, we also tested the degree of model overfitting to our dataset by adjusted estimates of discrimination and reclassification for model optimism using 1000 bootstrap samples.^39^

## Results

Among 18 231 parous women, 965 (5%) had an incident CVD event during follow-up (median 8.2 years, interquartile range 7.4–11.1 years) while 295 (2%) died from other causes. Non-fatal events comprised the majority of incident CVD events (97%) with 5% dying on the day of or within 30 days of the event. Thirty percent of women experienced at least one pregnancy complication based on birth registry records. After imputing pregnancy complications for births not captured in the registry, 39% of women had a history of pregnancy complications *in any pregnancy*. In age-standardized comparisons, women with a history of pregnancy complications based on birth registry records had higher systolic blood pressure (*P* < 0.001) and were more likely to smoke (*P* < 0.001), take anti-hypertensives (*P* < 0.001), have low HDL (*P* = 0.006), and have a family history of premature MI (*P* < 0.001) compared to women with no pregnancy complications (*Table 1*).

**Table 1:** Descriptive statistics of parous HUNT2 and HUNT3 participants by history of pregnancy complications at start of follow-up

	All study participants (*n* = 26 544^a^)	Did not experience a pregnancy complication^b^(*n* = 18 608^a^)	Experienced at least one pregnancy complication^b^(*n* = 7936^a^)
Age at HUNT exam in years, median (IQR)	52 (46–59)	52 (46–60)	51 (46–58)
Age-standardized^c^ risk factors from NORRISK 2 model			
Systolic blood pressure in mmHg, median (IQR)	128 (117–142)	127 (116–141)	130 (118–144)
Serum total cholesterol in mmol/L, median (IQR)	5.8 (5.1–6.6)	5.8 (5.1–6.6)	5.8 (5.1–6.6)
Current daily smoking	31%	29%	35%
Current anti-hypertensive use	13%	12%	18%
Low HDL-C^d^	37%	37%	39%
Family history of premature MI^e^	17%	16%	19%
Reproductive history			
Number of births			
1	8%	8%	7%
2	38%	39%	37%
3	34%	34%	35%
4+	19%	19%	21%
Pre-eclampsia in any pregnancy	5%	0%	17%
Gestational hypertension in any pregnancy	4%	0%	12%
Any preterm delivery	8%	0%	28%
Any small for gestational age delivery	18%	0%	62%

HUNT, the Nord-Trøndelag Health Study; HDL-C, high density lipoprotein cholesterol; IQR, interquartile range; MI, myocardial infarction.

a Women who participated in only HUNT2 (*n* = 3603) or HUNT3 (*n* = 6315) contributed one observation while women who participated in both HUNT2 and HUNT3 (*n* = 8313) contributed two.

b Pregnancy complications include pre-eclampsia, gestational hypertension, preterm delivery, and small for gestational age delivery.

c Risk factors standardized to the age distribution of the study population.

d Low HDL-C <1.3 mmol/L.

e First degree family member suffered MI before the age of 60 years.

### Model fit

The associations between established risk factors and CVD were similar to those reported in the original NORRISK 2 publication by Selmer *et al*.^20^ (Supplementary material online, *Table S3*). In unadjusted models including all pregnancy complications, pre-eclampsia, and SGA were independently associated with CVD (HR 1.96, 95% CI 1.44–2.65 for pre-eclampsia and HR 1.46, 95% CI 1.18–1.81 for SGA) (*Table 2*). After adjusting for the established risk factors only pre-eclampsia was associated with an increased rate of CVD (HR 1.60, 95% CI 1.16–2.17). Model fit was improved with the addition of pregnancy complications (Wald test *P* = 0.04).

**Table 2:** Hazard ratios for 10-year cardiovascular disease risk from Fine and Gray competing risk models comparing models with and without pregnancy complication history

Covariates	Unadjusted model for pregnancy complication history		Established risk factor model		Established risk factor model + pregnancy complication history^d^	
	HR^c^	95% CI	HR^c^	95% CI	HR^c^	95% CI
Age (per 1 year)			1.09	1.06–1.13	1.10	1.06–1.13
Age squared (per 1 year)			1.00	1.00–1.00	1.00	1.00–1.00
Systolic blood pressure (per 10 mmHg)			1.19	1.11–1.28	1.19	1.10–1.28
Serum total cholesterol (per 1 mmol/L)			1.12	1.06–1.18	1.12	1.06–1.18
Daily smoking (yes/no)			3.22	2.37–4.37	3.24	2.38–4.41
Systolic blood pressure × age			1.00	1.00–1.00	1.00	1.00–1.00
Daily smoking × age			0.98	0.97–1.00	0.98	0.97–1.00
Anti-hypertensives (yes/no)			1.41	1.21–1.66	1.38	1.16–1.64
Low HDL-cholesterol (yes/no)^a^			1.65	1.45–1.87	1.65	1.45–1.87
Family history of premature MI^b^			1.53	1.31–1.77	1.52	1.30–1.76
Pre-eclampsia in any pregnancy (yes/no)	1.96	1.44–2.65			1.60	1.16–2.17
Gestational HTN in any pregnancy (yes/no)	1.16	0.70–1.89			0.73	0.46–1.15
Any preterm delivery (yes/no)	1.13	0.85–1.51			0.98	0.73–1.31
Any SGA delivery (yes/no)	1.46	1.18–1.81			1.06	0.85–1.32

CI, confidence interval; HDL-C, high density lipoprotein cholesterol; HR, hazard ratio; HTN, hypertension; MI, myocardial infarction; SGA, small for gestational age.

a Low HDL-cholesterol: < 1.3 mmol/L.

b First degree family member suffered MI before the age of 60 years.

c CVD-specific hazard ratio from Fine and Gray competing risk model.

d Wald test for joint significance of all four pregnancy complications: *P* = 0.04.

### Calibration

The established risk factor model was well-calibrated both before (*P* = 0.23 for the null hypothesis of equal observed and predicted risk) and after (*P* = 0.26) the addition of pregnancy complications. A visual depiction of model calibration is provided in Supplementary material online, *Figure S1*.

### Discrimination

Inclusion of pregnancy complications in the established risk factor model led to a statistically significant but small 0.004 increase in the C-index (95% CI 0.002–0.006) (*Take home figure*). This small improvement in discrimination was driven mostly by the inclusion of pre-eclampsia (*Table 3*).

**Table 3:** Discrimination statistics comparing models with and without pregnancy complication history

Model	C-index	C-index difference from established CVD risk factor model	95% CI for C-index difference
Established CVD risk factors	0.789		
Established CVD risk factors + pre-eclampsia in any pregnancy	0.792	0.003	(0.001–0.005)
Established CVD risk factors + gestational HTN in any pregnancy	0.790	0.0006	(−0.0004 to 0.001)
Established CVD risk factors + any preterm delivery	0.789	0.0002	(0.0001–0.0002)
Established CVD risk factors + any SGA delivery	0.790	0.0003	(−0.0001 to 0.0008)
Established CVD risk factors + all pregnancy complications	0.793	0.004	(0.002–0.006)

CI, confidence interval; CVD, cardiovascular disease; HTN, hypertension; SGA, small-for-gestational age.

**Take home figure:**
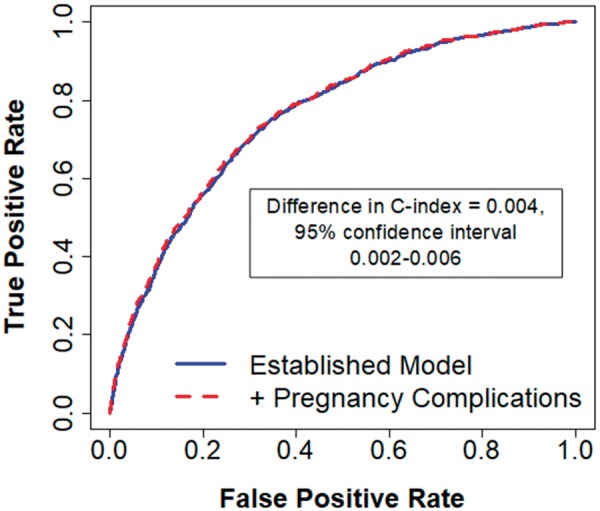
Pregnancy complications did not substantially improve 10-year cardiovascular disease risk prediction compared with an established model.

### Reclassification of clinical risk categories

Among the 965 women with an incident CVD event during follow-up, 38 were correctly reclassified into a higher risk category after the inclusion of pregnancy complication history while 17 were incorrectly reclassified into a lower risk category (*Table 4*). This lead to a marginal improvement in reclassification as measured by the NRI for events (0.02, 95% CI −0.002 to 0.04). Among the 25579 observations without a CVD event, 274 were incorrectly reclassified into a higher risk category while 375 were correctly reclassified into a lower risk category, which similarly resulted in a small improvement in reclassification [NRI for non-events 0.004 (95% CI 0.002–0.006)]. Overall, there was a small improvement in reclassification 0.02 (95% CI 0.002–0.05). No obvious improvements in reclassification were apparent when examining continuous predicted probabilities (Supplementary material online, *Figure S2*) and the IDI [−0.0002 (95% CI −0.001 to 0.0007)] did not indicate overall improvement on a continuous scale. Similar small improvements in reclassification were observed when comparing the established risk factor model to a model including pre-eclampsia history only (Supplementary material online, *Table S4*) and no improvement in reclassification was seen for the other pregnancy complications examined individually.

**Table 4:** **Reclassification of cardiovascular disease risk category after including pregnancy complication history**^a^^**,b**^

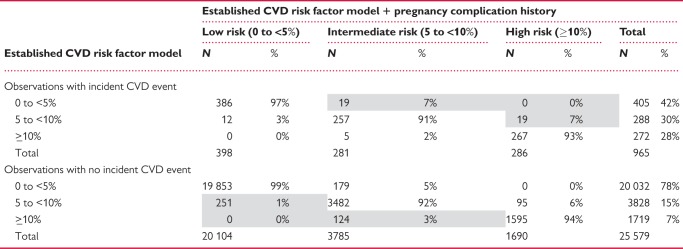

Net reclassification improvement (NRI) = 0.02 (95% CI 0.002 to 0.05), *P* = 0.04.

NRI for events = 0.02 (95% CI −0.002 to 0.04), *P* = 0.08.

NRI for non-events = 0.004 (95% CI 0.002 to 0.006), *P* < 0.001.

Integrated discrimination improvement (IDI) = −0.0002 (95% CI −0.001 to 0.0007), *P* = 0.65.

a Shaded areas represent improvements in reclassification after the addition of pregnancy complication history.

b This table includes observations with censoring prior to 10 years or follow-up longer than 10 years (median = 8.2 years of follow-up). Reclassification metrics explicitly take into account follow-up time (see Supplementary material online, Text *S^2^*) and cannot be directly calculated using numbers in this table.

### Additional analyses

Including additional variables for recurrent pregnancy complications and interactions between pregnancy complications did not lead to substantial improvements in prediction performance compared to models including only a single variable for history of each pregnancy complication (Supplementary material online, *Table S5*). Estimates were similar after adjusting for optimism (Supplementary material online, *Table S6*).

## Discussion

Addition of pregnancy complication history led to very modest to no improvements in an established cardiovascular risk prediction model recommended for clinical practice in Norway. We observed improvements in model discrimination and reclassification; however, differences were small in magnitude and unlikely to be clinically meaningful. Although pre-eclampsia and SGA were significant predictors of atherosclerotic CVD events in unadjusted models, only pre-eclampsia predicted an increased risk of CVD after adjusting for established risk factors.

### Comparison with current literature

Previous studies have examined the association between pregnancy complications and CVD^8–15^; however, significant associations do not necessarily translate into improved prediction.^40^ Only one previous study based on clinical data assessed prediction model performance after adding pregnancy complications and similarly found very modest to no improvement in CVD risk prediction.^18^ Our study expands on this work using a larger general population sample, covering a wider age range, using validated CVD events, and including preterm and SGA history in addition to hypertensive disorders of pregnancy. Our findings are also supported by a recent study based on self-reported data in female nurses, in which previous pre-eclampsia did not improve CVD risk prediction.^19^

The goal of this study was to examine the ability of pregnancy complications to predict CVD rather than their associations; however, it is worth noting that the magnitude of observed associations between pregnancy complications and CVD in our study were lower than those reported in previous studies, particularly for preterm, gestational hypertension, and SGA.^10–15^ This was true even compared to some Norwegian studies,^41–44^ although one study found similarly small estimates for preterm and SGA.^44^ Many previous studies were conducted using death registries and associations with pregnancy complications tend to be lower for non-fatal events^8^^,^^11^ which comprised the majority of events included in this study. Most previous studies also began follow-up time immediately after an index birth and there is some evidence that associations between pregnancy complications and CVD are substantially stronger at younger ages.^45^^,^^46^ In contrast, we started follow-up for CVD events after age 40 (mean = 52 years) to mimic clinical settings in which NORRISK 2 is used, which could also contribute to the modest associations seen in our study. In addition, few previous studies have adjusted for established risk factors included in prediction models.^8^^,^^12^ There is evidence that the association between HDP and CVD is mediated to a substantial degree by CVD risk factors,^47^ which may explain why including pre-eclampsia in the NORRISK 2 model did not substantially improve discrimination or reclassification.

### Prediction modelling choices

We chose the NORRISK 2 as our established risk factor model because it is currently recommended for clinical practice in Norway and was built using Norwegian data, including HUNT2 data among other sources. All decisions about how to model variables were made *a priori*, aligning with NORRISK 2 wherever possible, thus the risk of overfitting the prediction model to our dataset was low. We also confirmed that estimates were similar after adjusting for model optimism. We used identical definitions of model variables, CVD outcomes, and modelling techniques as the NORRISK 2 model with only one exception. NORRISK 2 includes separate variables for one compared to two or more family members with premature MI; however, this level of detail was not collected in HUNT3 so we used a single variable to summarize any family history of premature MI. The NORRISK 2 model is used exclusively in Norway and pregnancy complications may improve model fit more or less using other established risk prediction models. However, there is substantial overlap in the variables and methods used in NORRISK 2 compared to other commonly used CVD prediction models such as European SCORE,^48^ Framingham,^49^ or Pooled Cohort Risk Equations.^4^

Although there are many ways to model pregnancy complication history, we chose *a priori* to include a single variable indicating history of each pregnancy complication because it would be more feasible to implement a similar model in the clinical setting and aid in the interpretation of results. Still, models with alternative parameterizations of pregnancy complications yielded similar results, indicating little benefit to increasing model complexity.

### Limitations

While our study location in Nord-Trøndelag county is fairly representative of Norway,^24^ findings may not be generalizable to non-Nordic populations. For example, although smoking rates seen in our study were similar to the female Norwegian average during our study period,^50^ smoking prevalence is greater in Norway than most higher-income countries. Our study was also limited to parous women who made up about 90% of the population of women during the time period of this study.^51^ We would expect similar findings after including nulliparous women, although small improvements may be seen due to the association between parity and CVD.^52^^,^^53^ Another limitation of this study was the lack of data on gestational diabetes, which was likely underdiagnosed in the MBRN before 1988.^54^ The proportion of women who had ever experienced a pregnancy complication in this population (an estimated 39%) was higher than estimates from the United States (29%)^6^ but similar to a study from the United Kingdom (36%)^55^ One explanation for our high proportion of women with pregnancy complication history in this study is the relatively high fertility rate in Norway compared to other high-income countries.^56^ Another explanation is that the reference population used to identify birthweight percentiles by gestation length used a more recent sample of births in the MBRN. An advantage of using an external reference population is that our definition of SGA could be more easily recreated in other populations; however, increases in birthweights over time may have led to an overestimation of SGA deliveries.

### Strengths

Strengths of our study include the use of a general population sample of parous women and assessment of CVD risk factors during exams that reflect a realistic clinical scenario. In addition, 97% of the events used in this study were validated by study staff. Our linkage project provided a unique combination of rich clinical data, reproductive history, and follow-up for non-fatal and fatal events ideal for examining this research question.

### Clinical relevance and future directions

After adding pregnancy complications to the model, an additional 0.4% of women without events were correctly reclassified into lower risk categories while 2% of women with events were correctly reclassified into higher risk categories. In Norway, risk thresholds to initiative treatment to prevent CVD are age-specific but roughly align with thresholds used in this article,^21^ thus we would expect to see small improvements in appropriate treatment if pregnancy complications to CVD risk prediction models in clinical practice. While these benefits are small in magnitude, subsequent studies should evaluate whether greater improvements are seen for other populations, especially among younger women. Pregnancy complications occur early in life and may be useful for primary and primordial prevention in younger populations. Findings should also be validated in other populations, including non-Nordic countries.

## Conclusion

In this population-based, prospective cohort study, pregnancy complications (pre-eclampsia, gestational hypertension, preterm delivery, and SGA) led to only small improvements in 10-year CVD risk prediction for parous women, as measured by changes in model discrimination and reclassification. Although overall prevalence of at least one pregnancy complication was high in this population, pregnancy complications were not strong enough independent predictors of CVD after controlling for established risk factors to substantially improve prediction performance.

## Supplementary Material

Supplementary Data

Supplementary Material
