# Supplementary material for: Does pregnancy complication history improve cardiovascular disease risk prediction? Findings from the HUNT study in Norway
Source: Eur Heart J. 2018 Dec 27;40(14):1113–20. doi: 10.1093/eurheartj/ehy863 (PMC6451770; doi:10.1093/eurheartj/ehy863)
Supplement: Supplementary Data [file ehy863_supplementary_data.docx]

Supplementary Files – ONLINE ONLY

**Supplemental Table 1.** Description of established CVD risk factor measurement methods by HUNT exam

| **Risk Factor** | **HUNT2** | **HUNT3** |
| --- | --- | --- |
| Blood pressure | Staff measured blood pressure three times at 1-minute intervals after the person had come to rest using an automatic oscillometric method (Dinamap, Critikon, Florida), with cuff size adjusted to arm circumference. We defined systolic blood pressure as the mean of the second and third measurements. In HUNT3, 1,914 women were missing a third measurement due to staff shortages. Since the first measurement tends to be too low using Dinamap^1^, the second measurement was used in these cases. | |
| Cholesterol | Technicians at Levanger Hospital’s Central Laboratory assessed total cholesterol and high-density lipoprotein cholesterol (HDL-C), using the enzymatic colorimetric cholesterol esterase method with reagents from Boehringer Mannheim (Mannheim, Germany) using a Hitachi 911 Autoanalyzer. | Technicians at Levanger Hospital’s Central Laboratory measured total cholesterol using cholesterol esterase methodology and HDL-C using accelerator selective detergent methodology, all using reagents from Abbott (Abbott Ireland, Longford, Ireland; and Abbott Laboratories, Abbott Park, Illinois) using an Architect cSystems ci8200 |

^1^ Lund-Larsen PG. Blodtrykk målt med kvikksølvmanometer og med Dinamap under feltforhold - en sammenligning [Blood pressure measured with a sphygmomanometer and with Dinamap under field conditions – a comparison] Nor J Epidemiol. 1997;7:235–241.

**Supplemental Text 1.** Description of the cardiovascular event validation

From the electronic patient administrative system at the two primary hospitals in Nord-Trøndelag county, Levanger Hospital and Namsos Hospital (Nord-Trøndelag Hospital Trust), we obtained information on all study participants registered with at least one of the following cardiovascular diagnoses between September 1, 1987 (the first date of electronic recording) and April 24, 2015: ICD-9,402, 404, 410-414, 425, 427.5, 428, 430-438, 440-448; ICD-10, G45, I11, I13, I20-I25, I42, I46, I50, I60-I67, I71, I72, I74. For each identified patient, one of two experienced cardiologists (B.K., H.D.), who were unaware of the pregnancy history of the participants, examined the medical records to determine the first validated occurrence of a range of cardiovascular events, including myocardial infarction, cerebral infarction and intracranial hemorrhage. Myocardial infarction was diagnosed using ESC/ACCF/AHA/WHF criteria^1^. Cerebral infarction was diagnosed based on typical symptoms and signs combined with radiological evidence from CT or MRI scans. In some cases, the diagnosis of cerebral infarction was based on typical symptoms and signs in absence of radiological evidence, but only if CT or MRI scan had been performed, and no alternative explanation for the clinical presentation was found. Possible cerebrovascular events accompanying intracerebral malignancy, infection or inflammation were not included. Intracranial hemorrhage was classified based on radiological evidence as subarachnoid, intracerebral, subdural or epidural, and hemorrhage secondary to trauma or intracerebral malignancy was excluded. As stroke events in the present study, we included cerebral infarctions as well as intracerebral and subarachnoid hemorrhages.

1. Thygesen K, Alpert JS, Jaffe AS, Simoons ML, Chaitman BR, White HD. Third Universal Definition of Myocardial Infarction. *Circulation* 2012;**126**:2020–2035

**Supplemental Table 2.** ICD diagnosis codes for fatal cardiovascular endpoints

|  | **ICD-8 codes** | **ICD-9 codes** | **ICD-10 codes** |
| --- | --- | --- | --- |
| Fatal CHD | 410-414 | 410-414 | I20-I25 |
| Fatal stroke | 430-433 | 430, 431, 433-434 | I60, I61, I63 (excluding I63.6), I64 |

CHD=coronary heart disease

**Supplemental Text 2.** Additional description of statistical methods

a) Cluster-robust variance estimates

In the presence of clustered data, as in this manuscript where observations were clustered within women, naively estimated confidence intervals can be falsely narrow. To adjust for the correlation within women, we used cluster-robust estimates of the variance-covariance matrix. Robust variance estimation can be referred to by many different names, including sandwich or Huber-White^1,2^ estimation. In brief, the robust variance estimator can be thought of as a sandwich where the “bread” is the conventional estimator of variance and, in the context of survival analysis, the “meat” is typically an empirical variance estimator calculated using score residuals. The cluster-robust variance estimator is an extension of the robust variance estimator where the score residuals used to compute the “meat” of the sandwich are aggregated over identical ID values (in this case, aggregated across observations for the same woman). We implemented this method in SAS using the COVSANDWICH(AGGREGATE) option in PROC PHREG^3^.

b) Multiple imputation

Multiple imputation methods overview

To impute pregnancy complications for pregnancies either 1) missing from the registry or 2) with incomplete information in the registry, we used multiple imputation by fully conditional specification (FCS)^4^. FCS is also sometimes referred to as multiple imputation by chained equations (MICE or ICE) or sequential regression multiple imputation. In short, the FCS algorithm uses the following steps^5^:

1. Fill in random values for missing variables by sampling with replacement from the observed values
2. Starting with one variable (X_1_ for example), build a prediction model for the observed values of the variable based on all of the other variables
3. Impute missing values of X_1_ based on this prediction model, filling in the randomly chosen values with these imputed values
4. Repeat for all variables until all of the random values are filled in with imputed values

We used PROC MI in SAS to run the FCS algorithm. We chose to impute 20 times which means these steps were repeated 20 times resulting in 20 datasets with different imputed values. At 20 imputations, the efficiency in estimating beta coefficients is similar to the efficiency we would have seen had we used an infinite number of iterations^5^. In addition, we found little change in estimates when moving from 10 imputations to 20, suggesting that the increased computing time to produce additional imputations would have little to no effect on results.

In all Fine and Gray competing risk models reported in this paper that included pregnancy complications, we ran the model on each of the 20 datasets. Beta coefficients were calculated as the average across the 20 datasets and standard errors were corrected using Rubin’s rules^6^. We used PROC MIANALYZE in SAS to calculate corrected standard errors, confidence intervals, and p-values, including confidence intervals reported in Table 2 and the Wald test for model fit reported in the results section. All other prediction metrics (for calibration, discrimination, and reclassification) are based only on point estimates and not prediction model standard errors so they did not require any corrections for multiple imputation.

Imputation outcome variables:

We imputed pregnancy complications separately by birth order given the known differences in prevalence of pregnancy complications, particularly preeclampsia, by birth order. We imputed complications only for the first three pregnancies because information about later pregnancies was more complete. In addition, there were few observed pregnancy complications in fourth or later pregnancy (for example, only 56 observed preterm deliveries in fourth or later pregnancies) and imputation is less accurate with fewer observations. In total, we imputed 12 variables (i.e. 4 individual pregnancy complications across a woman’s first three pregnancies). After imputing the pregnancy complication status for each pregnancy separately, we aggregated across all pregnancies (observed and imputed) to indicate whether the women had any history of the complication of interest.

Imputation predictor variables

We included the following predictors in our imputation models: 1) maternal birth year, 2) whether the mother had a CVD event during follow-up, 3) whether the mother died from a non-CVD cause during follow-up, 4) all covariates included in the prediction model (age, age-squared, systolic blood pressure, serum total cholesterol, daily smoking, interaction terms for systolic blood pressure and age and daily smoking and age, antihypertensive use, low HDL-cholesterol, and family history of premature MI), and 5) pregnancy complications in other births. We assumed that the data were missing at random (MAR)^6^ after accounting for these variables. In other words, that the probability we had complete information on pregnancy complications did not depend on whether the woman had a pregnancy complication, after controlling for these variables. We think this is a reasonable assumption, particularly after controlling for birth year since the major reason for missing data was that births occurred prior to the start of the registry in 1967 which was more likely to happen for older women.

Imputation prediction method

We used the discriminant function method to classify women as having had a pregnancy complication or not (for example, preeclampsia in first pregnancy yes or no) for each imputation. We chose the discriminant function a priori since it has less issues with convergence than logistic regression; however, in sensitivity analyses, we found similar results using logistic regression.

Comparison of women with and without missing pregnancy information

Women with incomplete pregnancy complication history (i.e. women with at least one pregnancy that was either missing from the registry or who had incomplete information in the registry) were more likely to be born earlier and, thus, older at the time of the HUNT examinations. After age-standardizing, women with incomplete history had somewhat higher levels of blood pressure, serum total cholesterol, and smoking. These variables were all included in the imputation prediction models.

|  | **Complete pregnancy complication history (women=11,802 observations^a^=16,889)** | **Incomplete pregnancy complication history (women=6,429 observations^a^=9,655)** |
| --- | --- | --- |
| Maternal birth year, median (IQR) | 1952 (1948-1956) | 1943 (1938-1948) |
| Age at HUNT exam in years, median (IQR) | 49 (45-55) | 57 (51-64) |
| *Age-standardized ^b^ risk factors from NORRISK 2 Model* |  |  |
| Systolic blood pressure in mmHg, median (IQR) | 127 (116-140) | 129 (117-144) |
| Serum total cholesterol in mmol/L, median (IQR) | 5.7 (5.1-6.5) | 5.9 (5.1-6.7) |
| Current daily smoking | 30% | 32% |
| Current anti-hypertensive use | 13% | 13% |
| Low HDL-C^c^ | 37% | 37% |
| Family history of premature MI^d^ | 17% | 17% |

^a^ Women who participated in only HUNT2 (n=3,603) or HUNT3 (n=6,315) contributed one observation while women who participated in both HUNT2 and HUNT3 (n=8,313) contributed two.

^b^ Risk factors standardized to the age distribution of the study population

^c^ Low HDL-C: <1.3 mmol/L

^d^ First degree family member suffered MI before the age of 60 years

IQR = Interquartile range; HDL-C = high density lipoprotein cholesterol; MI = myocardial infarction

c) Prediction performance measures

Overview

This table summarizes the prediction performance measures used in this analysis.

| **Measure type** | **Measure assesses…** | **Specific measure used** |
| --- | --- | --- |
| Calibration | Equivalence between observed CVD risk and model-predicted CVD risk | Greenwood-Nam-D’Agostino test |
| Discrimination | Model ability to distinguish between CVD cases and non-cases | C-index and C-index difference |
| Reclassification | Improvement in CVD risk classification of the new model over the established model | Net reclassification improvement (overall and for events and non-events separately)  Integrated discrimination improvement |

Greenwood-D’Agostino-Nam test

The Greenwood-Nam-D’Agostino (GND) test^7^ is an extension of the Hosmer-Lemeshow test to situations with censored survival data. It is used to compare observed and expected (i.e. predicted) categories of CVD risk, typically categorized into deciles (G=10 in the formula below). The formula for the test statistic is:

$$\chi_{GND}^{2}\left( t \right)=\sum_{g=1}^{G} \frac{[KM_{g}\left( t \right)-\bar{p\left( t \right)}_{g}]^{2}}{Var(KM_{g}\left( t \right))}$$

Where:

$KM_{g}\left( t \right)=1-S_{g}(t)_{KM}$ is the Kaplan-Meier failure probability in the g^th^ decile at time t

$\bar{p\left( t \right)}_{g}$ is the mean predicted probability of failure for subjects in the g^th^ decile at time t

$Var\left( KM_{g}\left( t \right) \right)=(1-KM_{g}\left( t \right))^{2}\sum_{i|t_{i}\leq t} \frac{d_{i}}{n_{i}(n_{i}-d_{i})}$ is the Greenwood estimator of the variance of the probability of failure, where $d_{i}$ and $n_{i}$ are the number of failures and number at risk, respectively, at time $t_{i}$.

C-index

The concordance-statistic (C-statistic) is a frequently used measure of discrimination in logistic regression which reflects the area under the ROC curve (AUC). This measure is not appropriate for survival analysis, but the C-index^8^ is an extension of this same concept. We can interpret the C-index as the probability of concordance (agreement) between the predicted and observed survival times of any two subjects. For example, if subject A had a CVD event before subject B and subject A had a shorter predicted survival time, this would be counted as a concordant pair. If subject B had a shorter predicted survival time, this would be a discordant pair. Only pairs where at least one subject had an event are considered in the formula. The C-index is the percentage of all possible pairs who were concordant. Another way to express this is:

$$C=\frac{\pi_{c}}{\pi_{c}+\pi_{d}}$$

Where

$\pi_{c}=P(t_{i}<t_{j} and \hat{y}_{i}<\hat{y}_{j} or t_{i}>t_{j} and \hat{y}_{i}>\hat{y}_{j})$

$\pi_{d}=P(t_{i}<t_{j} and \hat{y}_{i}>\hat{y}_{j} or t_{i}>t_{j} and \hat{y}_{i}<\hat{y}_{j})$

$t_{i}$ and $t_{j}$ denote observed survival time for subjects *i* and *j*

$\hat{y}_{i}$ and $\hat{y}_{j}$ denote predicted survival time for subjects *i* and *j*

Across all pairs of subjects *i* and *j* where $t_{i}\neq t_{j}$

Net reclassification improvement

The net reclassification improvement (NRI) quantifies the improvement of a new model over an established model. In order to calculate the NRI, predicted probabilities must be divided into pre-established categories; for example, in this paper, we divided the 10-year risk of CVD into low (<5%), intermediate (5-<10%), and high (≥10%) risk groups. Improvements (i.e. correct reclassification) occur when subjects that had an event move “up” in their risk category under the new model and subjects that didn’t have an event move “down” in risk category. The basic formula is:

$$NRI=P\left( up | event \right)-P\left( down | event \right)+P\left( down | non-event \right)-P(up|non-event)$$

To extend the NRI to survival analysis^9^, this formula can be re-written using Bayes theorem to:

$$NRI=\frac{\left( P\left( event | up \right)-P\left( event \right) \right)\cdot P(up)+\left( P(event)-P\left( event|down \right) \right)\cdot P(down)}{P\left( event \right)\cdot(1-P\left( event \right))}$$

$P\left( event \right)$, $P\left( event | up \right)$, and $P\left( event|down \right)$ can all be estimated using the Kaplan-Meier approach while $P(up)$ and $P(down)$ are straightforward to estimate in the dataset.

We also calculated the NRI separately by event status, which are just components of the overall NRI formula broken out:

$${NRI}_{events}=P\left( up | event \right)-P\left( down | event \right)$$

$$=\frac{P\left( event | up \right)\cdot P\left( up \right)-P\left( event | down \right)\cdot P(down)}{P(event)}$$

$${NRI}_{non-events}=P\left( down | non-event \right)-P\left( up | non-event \right)$$

$$=\frac{(1-P\left( event | down \right))\cdot P\left( down \right)-(1-P\left( event | up \right))\cdot P(up)}{(1-P\left( event \right))}$$

Integrated discrimination improvement

The integrated discrimination improvement (IDI) is a continuous measure of reclassification.^10^ Improvements occur when the mean predicted probabilities (here, 10-year risk of CVD) increase under the new model for subjects that had an event and decrease for subjects that did not have an event. The IDI can be directly estimated from survival models.

$$IDI=\left( {\bar{\hat{p}}}_{new,events}-{\bar{\hat{p}}}_{new,non-events} \right)-({\bar{\hat{p}}}_{established,events}-{\bar{\hat{p}}}_{established,non-events})$$

Where, for examples, ${\bar{\hat{p}}}_{new,events}$ is the predicted probability of the event under the new model among subjects who had an event.

References

1. Huber PJ. The behavior of maximum likelihood estimates under nonstandard conditions. The Regents of the University of California; 1967.
2. White H. A Heteroskedasticity-Consistent Covariance Matrix Estimator and a Direct Test for Heteroskedasticity. *Econometrica* 1980;**48**:817–838.
3. Gharibvand L, Liu L. Analysis of Survival Data with Clustered Events. SAS Global Forum 2009. Paper 237-2009. https://support.sas.com/resources/papers/proceedings09/237-2009.pdf (10 September 2018).
4. Buuren S van. Multiple imputation of discrete and continuous data by fully conditional specification. *Stat Methods Med Res* 2007;**16**:219–242.
5. Carpenter J, Kenward M. Multiple Imputation and its Application. 1st ed. United Kingdom: John Wiley & Sons; 2013.
6. Rubin D. Multiple Imputation for Nonresponse in Surveys. New York: John Wiley & Sons; 1987.
7. Demler OV, Paynter NP, Cook NR. Tests of calibration and goodness-of-fit in the survival setting. *Statist Med* 2015;**34**:1659–1680.
8. Pencina MJ, D’Agostino RB. Overall C as a measure of discrimination in survival analysis: model specific population value and confidence interval estimation. *Stat Med* 2004;**23**:2109–2123.
9. Pencina MJ, D’Agostino RB, Steyerberg EW. Extensions of net reclassification improvement calculations to measure usefulness of new biomarkers. *Stat Med* 2011;**30**:11–21.
10. Pencina MJ, D’Agostino RB, D’Agostino RB, Vasan RS. Evaluating the added predictive ability of a new marker: from area under the ROC curve to reclassification and beyond. *Stat Med* 2008;**27**:157–172; discussion 207-212.

**Supplemental Table 3.** Comparison of NORRISK 2 model as reported in Selmer et al.^1^ to version used in this paper

| **Covariates^a^** | **NORRISK 2 Model as reported in Selmer et al. for women (N=35,267, endpoints=2,459)** | | | **Version of NORRISK 2 used in this paper  (N=18,231, endpoints=965)** | | |
| --- | --- | --- | --- | --- | --- | --- |
|  | **Beta** | **95% CI** | | **Beta** | **95% CI** | |
| Age (per 1 year) | 0.130 | 0.111 | 0.150 | 0.090 | 0.061 | 0.120 |
| Age squared (per 1 year) | -0.001 | -0.001 | -0.0002 | -0.000004 | -0.0007 | 0.0007 |
| Systolic blood pressure (per 10 mmHg) | 0.252 | 0.201 | 0.304 | 0.176 | 0.104 | 0.247 |
| Serum total cholesterol (per 1 mmol/L) | 0.072 | 0.033 | 0.111 | 0.113 | 0.058 | 0.169 |
| Daily smoking (yes/no) | 1.268 | 1.053 | 1.483 | 1.168 | 0.862 | 1.474 |
| Systolic blood pressure (per 10 mmHg) x age | -0.005 | -0.007 | -0.003 | -0.002 | -0.005 | 0.002 |
| Daily smoking x age | -0.025 | -0.033 | -0.016 | -0.019 | -0.034 | -0.004 |
| Anti-hypertensives (yes/no) | 0.192 | 0.091 | 0.293 | 0.347 | 0.190 | 0.504 |
| Low HDL-cholesterol^b^ (yes/no) | 0.324 | 0.239 | 0.408 | 0.499 | 0.372 | 0.627 |
| Family history of premature MI^c^: One family member | 0.254 | 0.142 | 0.366 |  |  |  |
| Family history of premature MI^c^: At least two family members | 0.549 | 0.321 | 0.777 |  |  |  |
| Family history of premature MI^c^ (yes/no) |  |  |  | 0.422 | 0.272 | 0.573 |
|  |  |  |  |  |  |  |
| Λ_0_(5)^d^ | 0.0010 |  |  | 0.0010 |  |  |
| Λ_0_(8)^d^ | 0.0017 |  |  | 0.0018 |  |  |
| Λ_0_(10)^d^ | 0.0023 |  |  | 0.0027 |  |  |

^a^ Age is centered at 40. Systolic blood pressure is centered at 120. Serum total cholesterol is centered at 4

^b^ Low HDL-cholesterol: < 1.3 mmol/L

^c^ First degree family member suffered MI before the age of 60 years

^d^ Λ0(5), Λ0(8) and Λ0(10) are baseline cumulative sub distribution hazards at 5, 8 and 10 years for age 40, systolic blood pressure 120 mmHg, serum total cholesterol 4 mmol/L, no smoking, HDL not low, no family history and not current user of antihypertensives.

^1^ Selmer R, Igland J, Ariansen I, Tverdal A, Njølstad I, Furu K, Tell GS, Klemsdal TO. NORRISK 2: A Norwegian risk model for acute cerebral stroke and myocardial infarction. Eur J Prev Cardiol 2017;24:773–782.

**A**
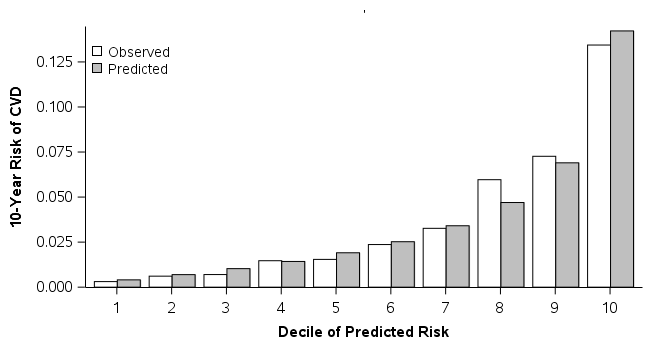


**B** **
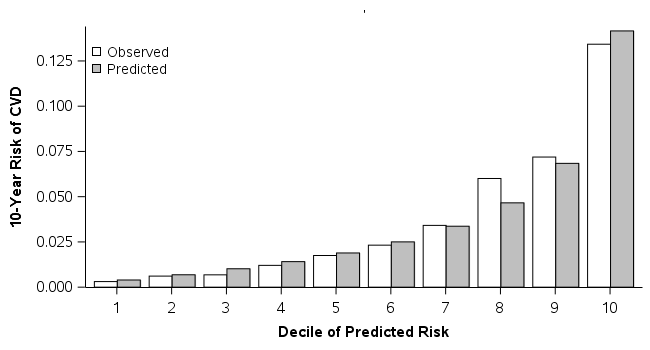
**

**Supplemental Figure 1.** Calibration plots for A) the established risk factor model and B) the established risk factor model with addition of pregnancy complications


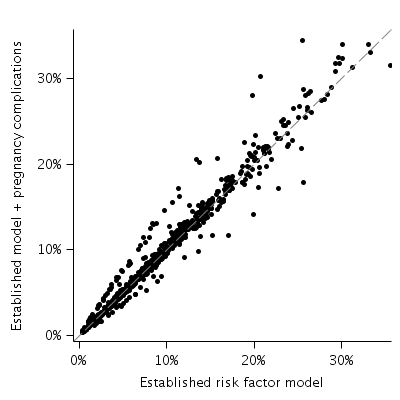

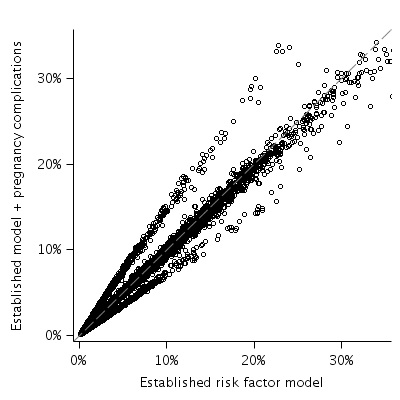
**A** **B**

Improved performance

Improved performance

**Supplemental Figure 2.** Reclassification plots of predicted probability before and after inclusion of pregnancy complications among A) observations where no CVD event occurred (n= 25,579), here observations below the dashed line indicate improved model performance and B) observations where a CVD event occurred (n=965), here observations above the dashed line indicate improved model performance. The cluster of women on or near the dashed line represent women without pregnancy complications, whose predicted probability remained similar in both models. The cluster of women well above the line represent women with preeclampsia, whose predicted probability increased and women well below the line represent women with gestational hypertension, whose predicted probability decreased after adding pregnancy complications to the model.

**Supplemental Table 4.** Reclassification of CVD risk category after including preeclampsia history ^a^

|  | Established CVD risk factor model + preeclampsia history | | | | | | | |
| --- | --- | --- | --- | --- | --- | --- | --- | --- |
|  | Low risk  (0-<5%) | | Intermediate risk  (5-<10%) | | High risk  (≥10%) | | Total | |
| Established CVD risk factor model | N | % | N | % | N | % | N | % |
| *Observations with incident CVD event* |  |  |  |  |  |  |  |  |
| 0-<5% | 388 | 97% | 17 | 6% | 0 | 0% | 405 | 42% |
| 5-<10% | 10 | 3% | 263 | 92% | 15 | 5% | 288 | 30% |
| ≥10% | 0 | 0% | 6 | 2% | 266 | 95% | 272 | 28% |
| **Total** | 398 |  | 286 |  | 281 |  | 965 |  |
|  |  |  |  |  |  |  |  |  |
| *Observations with no incident CVD event* |  |  |  |  |  |  |  |  |
| 0-<5% | 19,857 | 99% | 175 | 5% | 0 | 0% | 20,032 | 78% |
| 5-<10% | 223 | 1% | 3,520 | 93% | 85 | 5% | 3,828 | 15% |
| ≥10% | 0 | 0% | 103 | 3% | 1,616 | 95% | 1,719 | 7% |
| **Total** | 20,080 |  | 3,798 |  | 1,701 |  | 25,579 |  |

^a^ Shaded areas represent improvements in reclassification after the addition of preeclampsia history

Net reclassification improvement (NRI) = 0.02 (95% CI: -0.005, 0.04), p-value=0.11

NRI for events = 0.01 (95% CI: -0.002, 0.04), p-value=0.17

NRI for non-events = 0.002 (95% CI: 0.0006, 0.004), p-value=0.004

Integrated discrimination improvement (IDI) = 0.0001 (95% CI: -0.0005, 0.0008), p-value=0.83

**Supplemental Table 5.** Results from sensitivity analyses

|  | Main findings | | | Sensitivity analyses | | | | | |
| --- | --- | --- | --- | --- | --- | --- | --- | --- | --- |
|  | Established risk factor model + pregnancy complication history ^a^ | | | Established risk factor model + pregnancy complication history including recurrent pregnancy complications ^a,b^ | | | Established risk factor model + pregnancy complication history including interactions between pregnancy complications ^a,c^ | | |
|  | Estimate | 95% CI | p-value | Estimate | 95% CI | p-value | Estimate | 95% CI | p-value |
| *Model fit* |  |  |  |  |  |  |  |  |  |
| Wald test |  |  | 0.04 |  |  | 0.06 |  |  | 0.11 |
| *Calibration* |  |  |  |  |  |  |  |  |  |
| GND test ^d^ |  |  | 0.26 |  |  | 0.27 |  |  | 0.16 |
| *Discrimination* |  |  |  |  |  |  |  |  |  |
| C-index | 0.793 | (0.776, 0.807) | <0.001 | 0.794 | (0.777, 0.808) | <0.001 | 0.794 | (0.778, 0.809) | <0.001 |
| C-index difference ^e^ | 0.004 | (0.002, 0.006) | <0.001 | 0.004 | (0.002, 0.007) | 0.001 | 0.005 | (0.003, 0.007) | <0.001 |
| *Reclassification* ^e^ |  |  |  |  |  |  |  |  |  |
| NRI | 0.02 | (0.0002, 0.05) | 0.04 | 0.02 | (0.0002, 0.04) | 0.04 | 0.03 | (0.01, 0.05) | 0.001 |
| NRI events | 0.02 | (-0.002, 0.04) | 0.08 | 0.02 | (-0.004, 0.04) | 0.10 | 0.03 | (0.009, 0.04) | 0.01 |
| NRI non-events | 0.004 | (0.002, 0.006) | <0.001 | 0.004 | (0.002, 0.006) | <0.001 | 0.004 | (0.002, 0.006) | <0.001 |
| IDI | -0.0002 | (-0.001, 0.0007) | 0.65 | <0.0001 | (-0.0001, 0.0001) | 0.99 | -0.005 | (-0.001, 0.0004) | 0.29 |

^a^ Pregnancy complications include preeclampsia, gestational hypertension, preterm delivery, and small for gestational age delivery

^b^ Models include an additional term for each pregnancy complication indicating a history of two or more pregnancies with complication

^c^ Models include interaction terms between each pair of pregnancy complication history variables

^d^ Greenwood-Nam-D’Agostino test of calibration for censored survival data. Higher p-values indicate better model calibration

^e^ Compared to the established risk factor model

**Supplemental Table 6.** Comparing established risk factor model with and without pregnancy complication history^a^ after adjusting for optimism^b^

| Metric | Original estimate | Estimate adjusted for optimism |
| --- | --- | --- |
| *Discrimination* |  |  |
| C-index difference | 0.004 | 0.003 |
| *Reclassification* |  |  |
| NRI | 0.02 | 0.02 |
| NRI events | 0.02 | 0.02 |
| NRI non-events | 0.004 | 0.004 |
| IDI | -0.0002 | -0.0002 |

^a^ Pregnancy complications include preeclampsia, gestational hypertension, preterm delivery, and small for gestational age delivery

^b^ Optimism is a measure of the degree of model overfitting
